# A Rare Presentation of Rickets Mimicking Sacroiliitis: A Case Report and Literature Review

**DOI:** 10.7759/cureus.26033

**Published:** 2022-06-17

**Authors:** Rafal S Ali, Roua Abdulhussein, Michael Esrick, Mitali Sen

**Affiliations:** 1 Internal Medicine, Einstein Medical Center Montgomery, East Norriton, USA; 2 Internal Medicine, Al-Mustansiriyah University, College of Medicine, Baghdad, IRQ; 3 Rheumatology, Einstein Medical Center Philadelphia, Philadelphia, USA

**Keywords:** metabolic bone disease, inflammatory spondyloarthritis, sacroiliitis-like presentation, x-linked hypophosphatemic rickets, rickets

## Abstract

Hypophosphatemic rickets can cause a variety of bone and joint symptoms, one of its rare presentations is sacroiliac joint involvement, which may be mistaken for inflammatory spondylitis.

Here, we report the case of a 31-year-old African American woman who presented with a two-year history of lower back pain and morning stiffness, initially suspected to be due to inflammatory spondyloarthritis. Laboratory tests revealed negative inflammatory markers, normal serum calcium, vitamin D3, and parathyroid hormone levels; however, the alkaline phosphatase levels were elevated and serum phosphorus level was low. Magnetic resonance imaging (MRI) of the lumbosacral spine revealed mild widening of the sacroiliac joint with periarticular sclerosis with no signs of osteitis or bone marrow edema. Her condition was attributed to a known diagnosis of X-linked hypophosphatemic rickets affecting her sacroiliac joints. Her symptoms gradually improved after conservative treatment with physical therapy, nonsteroidal anti-inflammatory drugs, phosphate, and vitamin D supplementations.

Based on our literature review, we have come across only five rickets cases with similar presentations. Two patients had previously undiagnosed hypophosphatemic rickets at 15 and 35 years of age. One case was related to vitamin D-deficient rickets, and the final two cases were adult-onset vitamin D-resistant rickets misdiagnosed as ankylosing spondylitis.

Radiological signs of sacroiliac joint involvement in these cases include narrowing of the sacroiliac joints, fusion of the sacroiliac joints, subchondral hypointense signal changes, and chondral surface irregularities.

Vitamin D supplementation has significantly reduced the incidence of rickets; however, there are still cases of familial rickets that can present with a variety of symptoms, including signs and symptoms consistent with inflammatory spondylitis, which can be easily misdiagnosed or mistreated if this presentation is not recognized.

## Introduction

Rickets and osteomalacia are metabolic bone disorders that are characterized by decreased bone matrix mineralization. Rickets occurs before growth plates are fused and can be classified as calcipenic or phosphopenic/hypophosphatemic rickets.

Hypophosphatemic rickets (previously known as vitamin D-resistant rickets) involves mostly renal phosphate wasting with low serum phosphate levels, usually normal serum calcium, normal or mildly elevated parathyroid hormone (PTH) levels, elevated or sometimes normal alkaline phosphatase levels, normal serum 25-hydroxyvitamin D concentrations, and normal or slightly reduced serum 1,25-dihydroxyvitamin D concentrations. Most of these disorders are associated with high fibroblast growth factor 23 (FGF-23) levels.

X-linked, autosomal dominant, autosomal recessive hypophosphatemic rickets as well as hypophosphatemic rickets with hypercalciuria (a non-FGF-23-mediated mechanism) are hereditary variants of hypophosphatemic rickets that manifest with varying severity and age of onset. Malignancies can potentially cause acquired hypophosphatemic rickets.

The most common type of hereditary rickets is the X-linked inheritance with an incidence of 3.9 per 100,000 live births, this condition can result in many complications including limb deformities, deficient mineralization of the teeth, hyperparathyroidism, osteoarthritis, and pseudo-fractures. Given its nonspecific manifestations, the diagnosis and treatment are often delayed [1].

X-linked hypophosphatemic rickets can affect both males and females, with no significant differences in disease severity related to sex [2]. Musculoskeletal manifestations include slow growth, leg bowing, early osteoarthritis, enthesopathy, and osteophyte formation. In children, the growth plates surrounding the knee and the axial skeleton can appear dense on radiographic images, and an iliac bone scan can demonstrate osteomalacia and periosteocytic lesions [3,4].

Phosphate, calcitriol, and burosumab (monoclonal antibodies against fibroblast growth factor 23 (FGF-23)) are options for treatment. Patients with autosomal dominant hypophosphatemic rickets should be checked for iron deficiency and iron supplements should be given if needed, because low iron levels may contribute to the pathophysiology of this condition [5].

## Case presentation

We present the case of a 31-year-old African American woman with X-linked hypophosphatemic rickets who was referred to a rheumatology clinic complaining of progressive lower back and bilateral hip pain for two years. She was diagnosed with X-linked hypophosphatemic rickets when she was three-year-old and underwent corrective osteotomies of the left femur at the age of nine years. Her father was also diagnosed with rickets during childhood. She had given birth to a son two years ago following an uneventful pregnancy and was later diagnosed with hereditary rickets. She had used potassium phosphate when she was younger but had stopped using it five years before presentation on her own. Her medications upon presentation included over-the-counter vitamin D supplementation.

Her back and bilateral hip pain worsened in the months leading up to the presentation, to the point that she had to use a cane for ambulation. She also reported daily morning stiffness of the lower back lasting 30 minutes. She denied numbness or weakness in her lower extremities and there was no photosensitivity, skin rash, dry eyes, dry mouth, oral ulcers, or Raynaud phenomenon.

Physical examination revealed short stature with a measured height of 137 cm, paraspinal tenderness over the lumbar area, and a limited lower spine range of movement. Neurological assessment of both lower extremities was normal.

Laboratory tests showed erythrocyte sedimentation rate (ESR) of 10 mm/h (normal range: 0-29 mm/h), C-reactive protein (CRP) level of 3 mg/l (normal range: < 10 mg/l), negative human leukocyte antigen (HLA-B27), rheumatoid factor (RF), and anti-cyclic citrullinated peptide antibody testing within the normal range. Serum calcium was normal at 9.1 mg/dl (normal range: 8.5-10.2 mg/dl), and alkaline phosphatase levels were elevated ranging from 199 to 226 IU/l over the preceding 1 year (normal range: 44-147 IU/l). Serum 25-hydroxy vitamin D level was 63 ng/ml (normal range: 20-100 ng/ml). Serum phosphorus level of 2 mg/dl (normal range: 2.8-4.5 mg/dl), PTH level was normal at 53 pg/ml (normal range: 10-55 pg/ml). A 24-hour urine phosphorus level was normal at 1.074 g/24 h (normal range: 0.4-1.3 g/24 h). A 24-hour urine calcium level was low at 65 mg/day (normal range: 100-300 mg/day). Complete blood count, liver function, and renal function test results were within normal ranges. Thyroid-stimulating hormone (TSH) was within the normal range at 1.55 mIU/l (normal range: 0.5-5 mIU/l).

**Figure 1:**
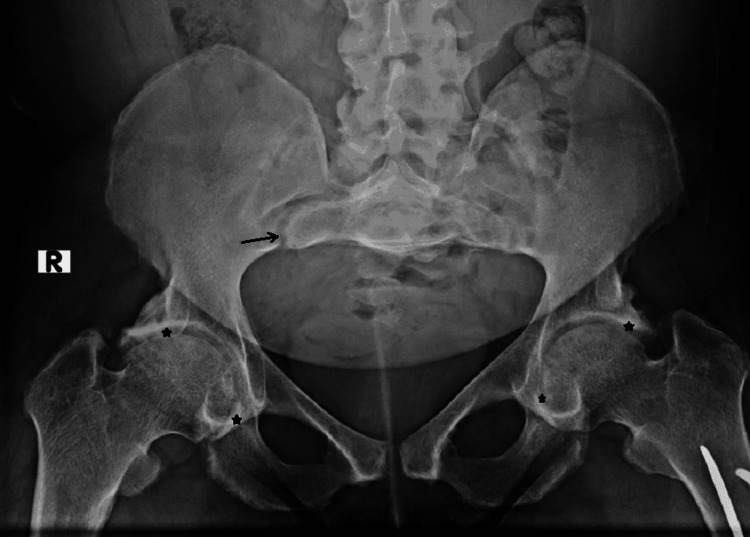
Plain film radiography of the pelvis showed moderate bilateral hip osteoarthritis (stars) and chronic erosive changes in the sacroiliac joints (arrow).

**Figure 2:**
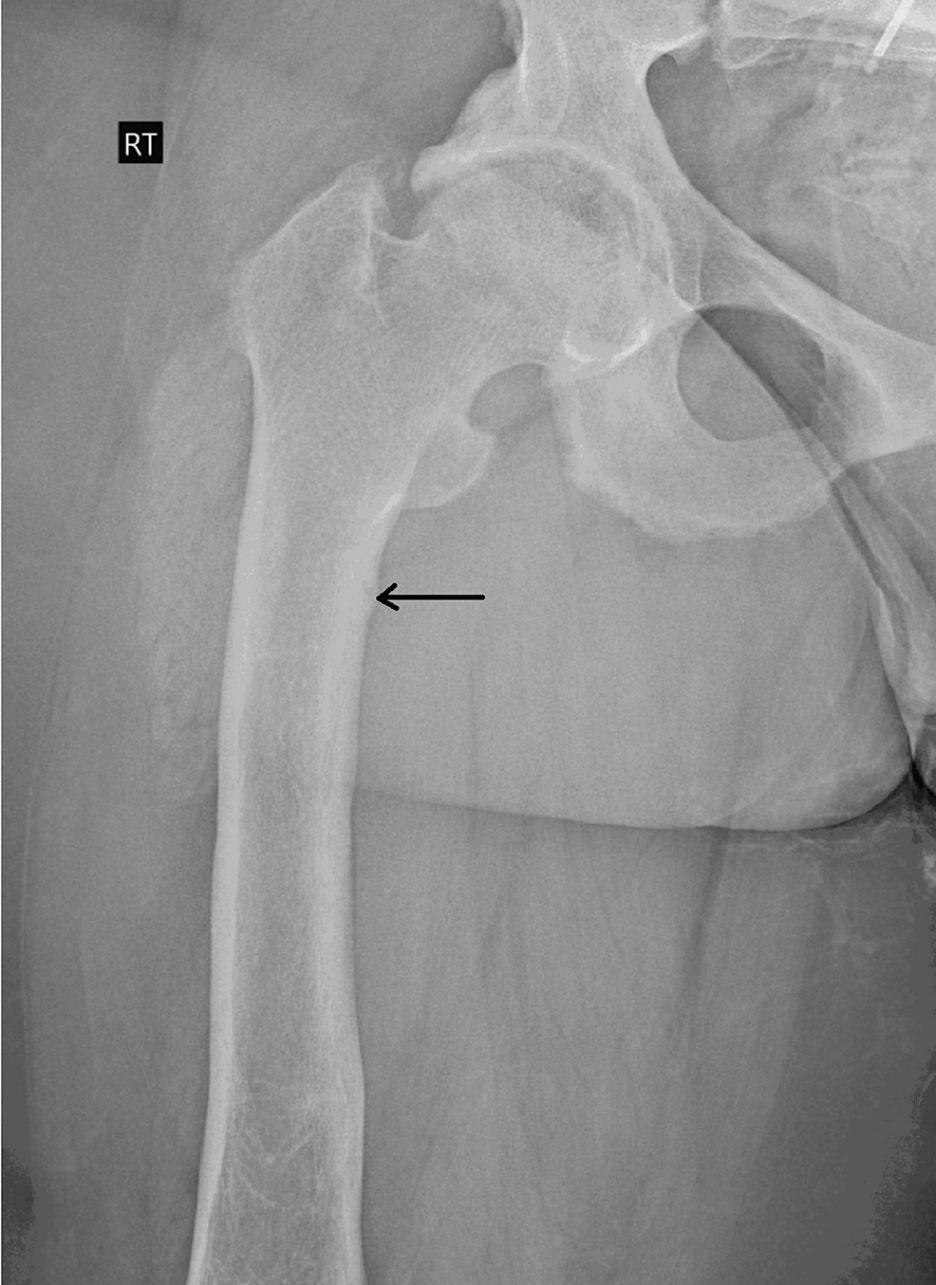
Transverse lucency across the medial cortex of the right proximal femur representing a Looser zone (arrow).

**Figure 3:**
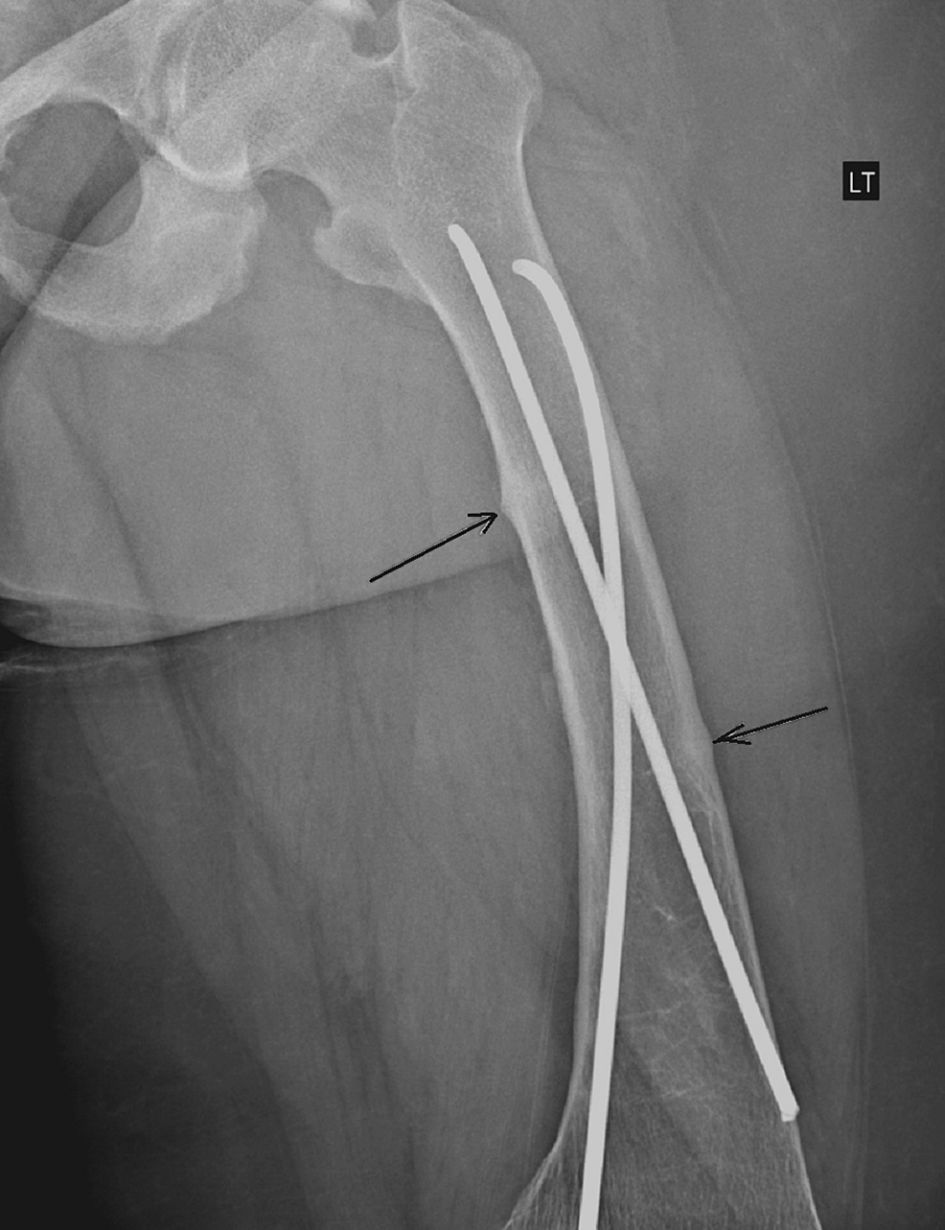
Stable fixation of the left femur with two intramedullary rods, and a healed fracture along the medial aspect of the proximal shaft as well as lateral aspect of the midshaft of the left femur (arrows).

**Figure 4:**
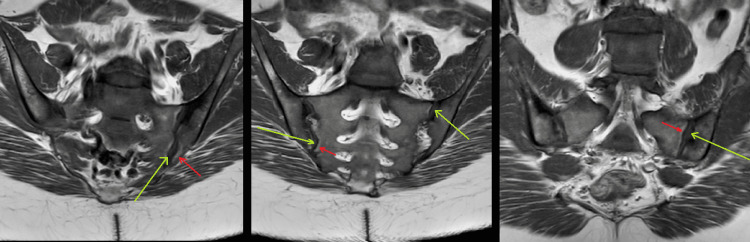
MRI of the sacrum revealed mild widening of the sacroiliac joints (long arrows) with periarticular sclerosis (short arrows).

Plain film radiography of the pelvis showed moderate bilateral hip osteoarthritis and chronic erosive changes in the sacroiliac joints (Figure 1). Transverse lucency was observed across the medial cortex of the right proximal femur, which possibly indicated a Looser zone or insufficiency fracture Figure 2). Radiography of the left femur showed stable fixation of the left femur with two intramedullary rods and a healed fracture along the medial aspect of the proximal shaft and the lateral aspect of the midshaft of the left femur (Figure 3). Magnetic resonance imaging (MRI) of the sacrum was later performed and revealed mild widening of the sacroiliac joints with periarticular sclerosis but no evidence of osteitis (Figure 4).

These findings indicate the presence of degenerative changes. Notably, the patient had undergone lumbosacral MRI two years prior to presentation, which was normal.

She was evaluated by orthopedic surgery for bilateral hip osteoarthritis, at which time she received bilateral hip corticosteroid injections. This resulted in a two-month pain-free period. Given the negative inflammatory spondylitis workup, her hip problem was subsequently attributed to accelerated osteoarthritis secondary to rickets. Changes in the sacroiliac joint were attributed to degenerative changes of rickets. Her lower back discomfort improved with physical therapy, phosphorus, and vitamin D supplements; however, she still experienced bilateral hip pain. At the time of this case report, the patient was being evaluated by orthopedic surgery for bilateral total hip arthroplasty.

## Discussion

Deficient bone mineralization can result in rickets and/or osteomalacia. Rickets occurs before the growth plates are closed and can present with delayed closure of fontanelles, bone deformities, and short stature. Radiographic signs include widening of the epiphyseal plate, long bone deformities, pathological fractures, and pseudo-fractures.

Although vitamin D treatment has reduced the incidence of rickets, familial cases remain. Our patient is known to have X-linked hypophosphatemic rickets since early childhood, with short stature and long bone deformity. Laboratory findings were consistent with hypophosphatemic rickets: normal serum calcium, decreased serum phosphorus concentration, normal PTH level, mild to moderately elevated alkaline phosphatase level, and normal vitamin D concentration. She had radiologic findings consistent with hypophosphatemic rickets, including accelerated and premature osteoarthritis of her bilateral hip joints, and mild widening of the sacroiliac joints with periarticular sclerosis. MRI of the sacroiliac joints did not show the typical features of inflammatory sacroiliitis (bone marrow edema, osteitis, synovitis, and bone erosions).

Although initially she was thought to have possible inflammatory sacroiliitis, her symptoms were in fact secondary to rickets bones, and joint changes.

According to our literature review, we found only five rickets cases presenting with a sacroiliitis-like picture. Most patients were female, with a mean age of 23.75 years. These cases presented with low back pain, normal inflammatory markers, and imaging findings including widening, narrowing, fusion of the sacroiliac joint, multiple erosions of the iliac crest, and sclerotic changes in the lumbar spine. The five cases were diagnosed as follows: two cases with hypophosphatemic rickets, one patient was diagnosed with a vitamin D deficiency and two cases were diagnosed with adult-onset vitamin D-resistant rickets with associated benign mesenchymal tumors (Table 1) [6-9].

**Table 1:** List of rickets cases presenting with inflammatory spondylitis-like picture. SIJ: sacroiliac joint; PTH: parathyroid hormone

Case	Diagnosis	Age years	Sex	Presentation	Imaging	Inflammatory markers	Laboratory	Reference
1	X-linked hypophosphatemic rickets	31	Female	Low back pain	Widening of SIJ	Negative	Hypophosphatemia	Our case
2	Hypophosphatemic rickets	15	Female	Low back pain	SIJ involvement on X-ray	Negative	Low phosphate, normal vitamin D, and calcium	[6]
3	Hypophosphatemic rickets	35	Female	Low back pain	Multiple erosions of the iliac crest, narrowing of the sacroiliac joints, sclerotic changes in the lumbar spine	Negative	Low phosphorus and vitamin D levels, normal Ca level	[7]
4	Vitamin D deficiency rickets	14	Female	Low back pain	MRI findings indicative of sacroiliitis	Negative	Low calcium, phosphorus, and vitamin D levels. Elevated PTH	[8]
5	Adult-onset vitamin D-resistant rickets with associated benign mesenchymal tumors	Adult	Not reported	Low back pain	Fused sacroiliac joints	Negative	Not reported	[9]
6	Adult-onset vitamin D-resistant rickets with associated benign mesenchymal tumors	Adult	Not reported	Low back pain	Not reported	Negative	Not reported	[9]

## Conclusions

Rickets is a metabolic bone disease that can present with a wide range of symptoms including bone and joint involvement. Axial involvement, especially sacroiliac joint involvement, can be misdiagnosed as an inflammatory rheumatic disease; therefore, it is important for clinicians to consider metabolic diseases in the differential diagnosis when evaluating such individuals.

Laboratory tests and imaging modalities (plain radiography and MRI) can help to differentiate both entities. The absence of osteitis or bone marrow edema should warrant the clinicians to investigate other etiologies including rickets/osteomalacia.
